# Medical Graduates

**Published:** 1830

**Authors:** 


*Medical Graduates* During the past spring, the degree of Doctor of Medicine has been conferred by the University of Pennsylvania on 125 gentlemen ; by the Transylvania University 71 ; by the Medical College of South Carolina 55 ; by the University of Maryland 53 ; by the Medical College of Ohio 26.

